# The potential moderating role of living in a conflict area on the link between classroom psychosocial stressors, perceived stress and change in anxiety symptoms in Israeli school children

**DOI:** 10.1002/jcv2.12092

**Published:** 2022-09-16

**Authors:** Pia Behnsen, Pol van Lier, Kinneret Levavi, Joanne Marieke Buil, Anja Huizink, Susanne Koot, Naama Atzaba‐Poria

**Affiliations:** ^1^ Department of Clinical, Neuro‐ and Developmental Psychology Vrije University Amsterdam Amsterdam Public Health Research Institute Amsterdam The Netherlands; ^2^ Department of Psychology Ben Gurion University of the Negev Be'er Sheva Israel; ^3^ Radboud University Medical Center Nijmegen The Netherlands

**Keywords:** anxiety problems, children, perceived stress, school stressors

## Abstract

**Background:**

Perceived stress associated with relational victimization from peers and conflictual relations with the teacher have been linked with the development of anxiety symptoms in children. Living in conditions of ongoing stress from the broader environment has also been related to anxiety symptoms in children. In this study, we examined (a) the indirect effect between classroom psychosocial stressors (i.e., relational victimization and conflictual relationships with teachers), perceived stress and anxiety symptom development, and (b) whether this indirect effect was stronger for children living in a high threat region than for children living in a lower threat region.

**Methods:**

Children participating in the study attended elementary school either in a high threat of armed conflict region (15 s to hide in bomb shelter when alarm sounds, *n* = 220) or in a lower threat of armed conflict region (60 s to hide in a bomb shelter when alarm sounds, *n* = 188) in Israel. Children were first assessed on conflictual relationships with teachers and peers, subjectively perceived stress and anxiety in 2017 (*T*_0_; *M* age = 10.61 years, *SD* = 0.78; 45% boys) and re‐assessed (*T*_1_) 1 year later, in 2018.

**Results:**

Perceived stress mediated the association between classroom psychosocial stressors and anxiety development. No moderation by threat‐region was found in this indirect effect. However, the association between perceived stress and anxiety development was only significant for children in the high threat region.

**Conclusion:**

Our study suggests that threat of war conflict amplifies the association between perceived stress and the development of anxiety symptoms.

## Graphical abstract

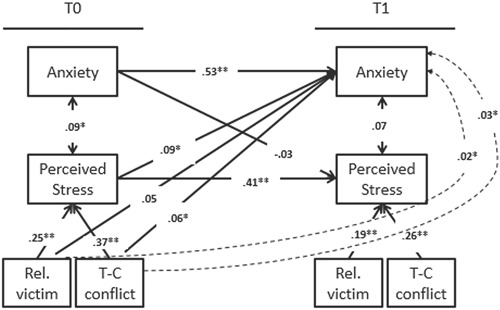

Our study suggests that threat of war‐conflict plays a significant role in the relationship between perceived stress and anxiety symptoms and serves as an environmental risk factor for children's development of anxiety problems.

**Key points**

- moderating effect of living in a conflict environment on the predictive link between perceived stress and anxiety symptoms.
- link perceived stress to anxiety symptoms only significant in Israeli children living in a high threat of armed conflict region.
- armed conflict plays an environmental risk factor for children's development of anxiety problems.
- findings urge preventive interventions against the onset of anxiety problems for high‐risk children.

## INTRODUCTION

Anxiety in children may develop from a complex set of risk factors. While anxiety is one of the most pervasive and common mental disorders, risk factors for the development of anxiety are understudied. There is strong evidence that exposure to environmental stressors and psychosocial stressors early in life can influence the development of anxiety (Racine et al., 2021). We will focus on classroom psychosocial stressors in Israeli children who live in armed conflict zones, which has been linked to the development of anxiety symptoms (Evans & English, 2002; Hammen, 2005; Kar, 2009; Slattery et al., 2012).

One important psychosocial risk factor for anxiety in childhood is a negative relationship with teachers and classroom peers. These psychosocial stressors within the classroom environment constitute important childhood risk factors for the development of perceived stress as well as subsequent anxiety problems (van der Wal et al., 2003; Veenstra et al., 2005; Wang & Fletcher, 2017). Furthermore, particularly self‐reported perceived stress has been associated with increased anxiety symptoms in European and US American childhood samples (Dieleman et al., 2015; Monk et al., 2001; Pittig et al., 2013) and also in Israeli adolescents (Dimitry, 2012). This may indicate that the association between psychosocial classroom stressors and the development of anxiety symptoms is partially explained via children's subjectively perceived stress levels.

Another important psychosocial risk factor for anxiety is stress derived from the living environment, particularly the (threat of) being exposed to conflict and violence (Bar‐Haim et al., 2010; Dubow et al., 2012). The effects of psychosocial risk factors in the classroom, the conflictual nature of relationships with peers and teachers, on the development of anxiety via perceived stress might be amplified in the context of conflict and threat (Cohen & Eid, 2007). Therefore, the first aim of this study was to test whether social stressors at school (i.e. conflictual relationships with teachers and peers) are associated with Israeli children's levels of perceived stress, and – in turn – with the development of anxiety symptoms. Our second aim was to examine whether the indirect association between relational victimization and conflictual relationships with the teacher, perceived stress and anxiety development was stronger for children growing up in a high threat (of armed conflict) region, compared to children growing up in a lower threat region of Israel.

### Allostatic load‐stressors across multiple contexts

The cumulative burden of chronic stressors, such as ongoing political conflict, is referred to as allostatic load. Ideally, individuals with high allostatic load show higher levels of self‐rated perceived stress (Tomba & Offidani, 2012), indicating that subjectively perceived stress levels might be a marker of allostatic load. Furthermore, high allostatic load is associated with poorer mental health among children and adolescents (Evans & English, 2002). Thus, children who experience a combination of psychosocial stressors at school (conflictual relationships) as well as environmental stressors (living in a threat region) might experience a higher allostatic load (perceived stress) and therefore an increased risk of developing anxiety problems.

### The influence of negative classroom social experiences on anxiety symptoms

An important psychosocial factor to investigate is the classroom, in which children spend a lot of time during the day. Childhood relational peer victimization comprises deliberate and repeated behavior intended to actively exclude a child from social activities, or to threaten or damage a victim's relationship with peers (Crick & Bigbee, 1998). Furthermore, a conflictual teacher‐child relationship refers to a pattern of negativity, and lack of support of the teacher to the child and is distressing for children (Hatfield & Williford, 2017). Conflictual relationships in the classroom have been linked to perceived stress (Wang & Fletcher, 2017). In turn, perceived stress has been associated with higher levels of anxiety symptoms in European and US American childhood samples (Dieleman et al., 2015; Monk et al., 2001) and also in Israeli adolescents (Dimitry, 2012).

### The influence of Gaza vicinity on the indirect association between classroom psychosocial stressors, perceived stress and anxiety

Another important contextual factor to consider is the living environment, particularly the exposure to conflict and violence. Children growing up in the Gaza vicinity experience frequent alarm soundings, sometimes multiple times per day (Cohen & Eid, 2007; Doeland, 2012) and have only 15 s to seek shelter (Lahav et al., 2019). Children living in area's further away from the Gaza strip may be incidentally exposed to rocket alarms and have longer period (e.g., 1 minute or more) to seek shelter (Besser & Neria, 2012). People living in geographical closeness to the Gaza strip are at risk of suffering death or injury due to the rocket attacks, experience a lack of control or symptoms of post‐traumatic stress disorder (Bar‐Haim et al., 2010; Barber & Schluterman, 2009; Cohen & Eid, 2007; Doeland, 2012; Dubow et al., 2012; Israeli Ministry of Foreign Affairs, 2015).

The link between children's perceived stress associated with psychosocial stressors within the classroom context and the development of anxiety symptoms might be more amplified among children living in the Gaza vicinity compared to children living further away from the Gaza strip. This is because being exposed to classroom psychosocial stressors in combination with being exposed to a stressful living environment would likely result in more allostatic load, as expressed by children's subjectively perceived stress. Related results would emphasize that teachers and school counselors should be particularly aware of the effects of exposure to school social stressors, perceived stress and anxiety in children in conflict regions.

### The aim of the current study

Our first aim was to test whether psychosocial stressors in children's classroom environment (i.e., relational victimization and conflictual relationships with teachers) were associated with changes in anxiety symptoms 1 year later, via perceived stress. Our second aim, on moderation by threat‐region, was to test whether the association between relational victimization/conflictual teacher‐child relationships, perceived stress and anxiety symptoms was stronger for children living in the high threat region than for children living in the lower threat region. To this end, children (aged 9–11 years at first assessment) attending mainstream elementary schools in Israel were followed across one school year.

## METHODS

### Sample

Data were collected in seven mainstream elementary schools in Israel. Of the total sample of 530 children, *n* = 269 children of four schools (18 classrooms) were living in a midsized city in the direct vicinity of Gaza (Sderot; 15 s to bomb shelter when alarm sounds, high threat region), and *n* = 261 children of three schools (21 classrooms) were living in a midsized city (Be'er Sheva; 60 s region; lower threat region). Children were first assessed in 2017 (*T*_0_; grades 4–5, *M* age = 10.61 years, *SD* = 0.78 years; 45% boys) and re‐assessed in 2018 (*T*_1_; grades 5–6, *M* age = 11.12 years, *SD* = 0.96 years). In the seven schools that participated in this study, these schools with participating children were randomly selected out of 20 schools in total. These schools were asked to participate in the study as they are representative of the population in the regions we included in the study. Only children from Jewish schools were assessed in the current study. However, the children were representative of the general population in the addressed regions. The questionnaires were completed anonymously, in Hebrew. The questionnaires were translated into Hebrew from the original Dutch or composed in Hebrew specifically for this study.

Only children with valid data on anxiety symptoms at *T_1_* (main outcome variable) were included in the present study, resulting in 408 children (lower threat region *n* = 188, high threat region *n* = 220). Non‐included children did not differ from included children on gender and age, or on *T_0_* scores of anxiety, perceived stress, relational victimization of conflictual teacher‐child relationship (all *p*'s > 0.05). However, excluded children were more likely to come from the lower threat region when compared to the high threat region; χ^2^(1) = 15.507, *p* < 0.001.

### Procedure

Ethical approval was obtained from the Israeli Ministry of Education's ethic committee as well as from the Helsinki committee of Soroka medical center in Israel. Parents of participants provided written informed consent after procedures were fully explained. Self‐ and teacher‐reported data were collected during a school day at school at both waves.

### Measures

**Anxiety symptoms**. Teacher reported symptoms of anxiety were assessed with the Problem Behavior At School Interview (PBSI) (Erasmus MC, 2000). The PBSI is a 42‐item instrument that assesses internalizing and externalizing symptoms in children as perceived by teachers. The 5‐item anxiety scale was used, items included “This child is nervous or tense”. Teachers rated children on a 5‐point Likert scale, ranging from 0 [*never*] to 4 [*often*]. Sum scores were used. Cronbach's Alpha was 0.84 at *T*_0_ and 0.79 at *T*_1_. Student's anxiety measured with the PBSI was associated with other outcomes such as peer rejection, self‐concept and teacher‐child interaction in previous published studies (e.g. Breeman et al., 2015). The PBSI has been shown to have adequate convergent validity, and sensitivity to change was demonstrated in a preventive intervention study (Spilled et al., 2013).

**Perceived Stress** was assessed with the Maastricht Stress Questionnaire, which is a self‐report questionnaire measuring physical and psychological stress symptoms experienced during the past week (Kraag et al., 2009). The 10‐item psychological scale was used, items included general perceived stress items such as “I find it hard to calm down”. One item of the scale referred to stress in the classroom “How often do you have the feeling that the other children in the classroom are too much for you?”. Children rated on a 4‐point Likert scale, ranging from 0 [*never*] to 3 [*often*]. Sum scores were used. Cronbach's alpha was 0.89 at *T*_0_ and 0.76 at *T*_1_. Perceived stress measured with the Maastricht Stress Questionnaires was previously associated with other measures such as stress awareness (Kraag et al., 2009).

**Psychosocial stressor relational victimization** was assessed with the Social Experiences Questionnaire Self‐Report (SEQ‐S; Grotpeter & Crick, 1996). The 5‐item relational victimization scale was used, items included “Are you excluded (e.g., from games) if a classmate is angry at you ‐ by that angry child?”. Children rated on a 5‐point Likert scale, ranging from 0 [*not true*] to 4 [*very true*]. Sum scores were used. Cronbach's Alpha was 0.79 at *T*_0_ and 0.76 at *T*_1_. Relational victimization measured with the Social Experience Questionnaire was previously linked to meaningful measures such as anxiety and depressive symptoms (Dempsey & Storch, 2008; Storch, Crisp, et al., 2005).

**Psychosocial stressor conflictual teacher‐child relationships** was assessed with The Young Children's Appraisals of Teacher Support (Y‐CATS; Mantzicopoulos, 2005). The Y‐CATS assesses children's perceptions of the relationship with their teacher. The 10‐item conflict subscale was used, items included “My teacher gets angry with me”. Every item has two answer possibilities, 0 [*true*] and 1 [*not true*]. Sum scores were used. Cronbach's alpha was 0.76 at *T*_0_ and 0.74 at *T*_1_. Self‐reports of conflictual teacher‐child relationships assessed with the Young Children's Assessment on Teacher Support were previously linked to children's problem behavior (Mantzicopoulos, 2005; Spilled et al., 2010).

**Environmental stress region** was dummy coded with 0 = lower threat region and 1 = high threat region.

### Control variables

**Sex** and **age** were assessed with self‐report, sex was coded with 0 = female, 1 = male.

**Family size** was assessed by asking children how many children live in their family. Increased family size has been related to heightened perceived stress among children (Shaw et al., 1994), thus we included family size as a control variable.

### Statistical analysis

**FIGURE 1:**
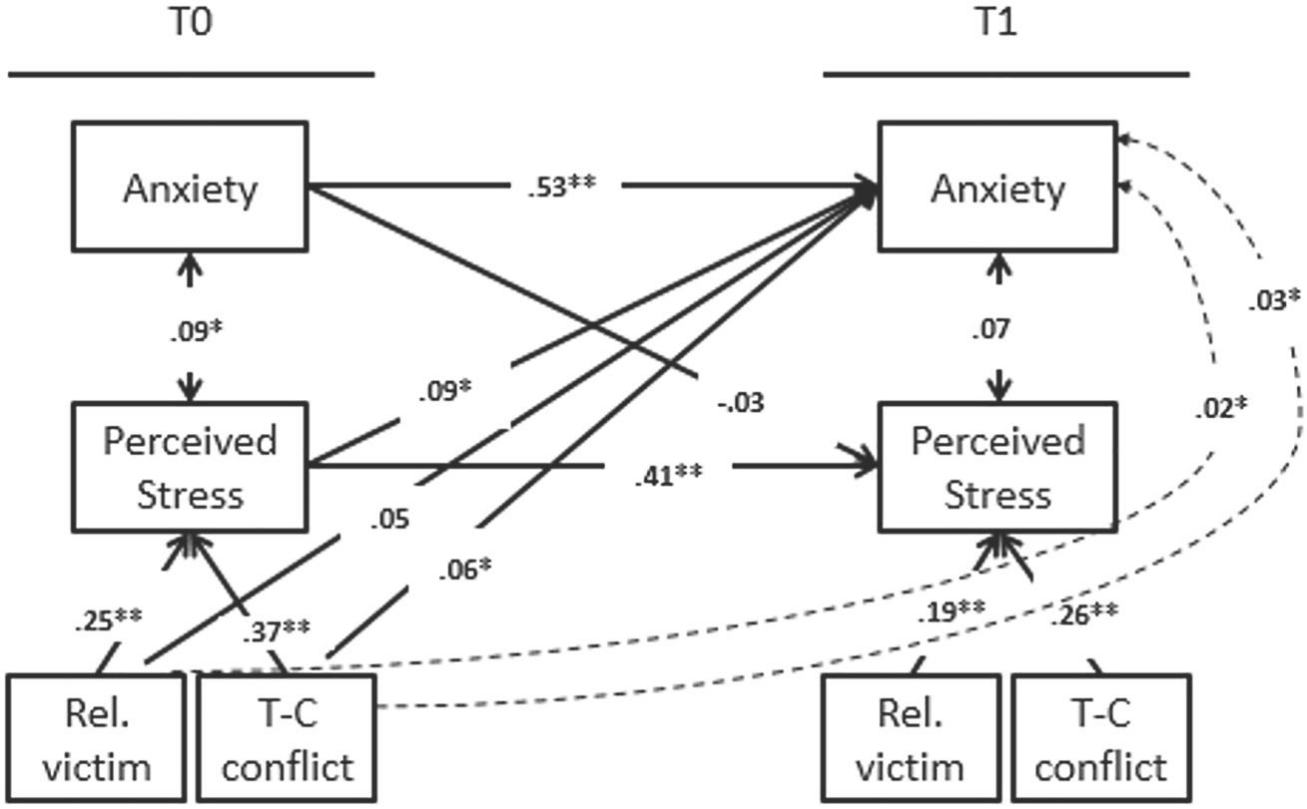
Overview of path model. Entries in single headed arrows reflect path estimates (standardized regression coefficients) across the high and lower threat region. Entries in double headed arrows reflect correlations coefficients. Entries in the dotted arrow reflect the indirect effect. Rel. victim, Relational victimization; T‐C conflict, Teacher‐Child Conflict. Overall sample *n* = 408. CFI = 0.95, RMSEA = 0.04 and SRMR = 0.04. *p < 0.05, **p < 0.01.

Our study hypotheses were tested using autoregressive cross‐lagged path models (see Figure 1 for the full model, including the indirect effects). We first tested whether classroom *T*_0_ psychosocial stressors predicted anxiety development from *T*_0_ to *T*_1_, via *T*_0_ perceived stress, without moderation by stress‐region_._ In step I, we tested whether relational peer victimization and teacher‐child conflict predicted relative change in the level of anxiety symptoms 1 year later. In step II, we added the mediator perceived stress and tested an indirect effect from *T*_1_ relational victimization/teacher‐child conflict to change in anxiety symptom levels from *T*_0_ to *T*_1_, via *T*_0_ perceived stress. Path estimates were controlled for sex, age, family size, and threat region.

Second, we tested for moderation by threat‐region using multiple‐group models (lower vs. high threat region), using the Satorra‐Bentler Chi Square Difference Test (Satorra & Bentler, 2001). In step III, we tested for moderation of main effects. To this end, we tested whether the paths from relational victimization/teacher‐child conflict, respectively, to subsequent anxiety differed between the two groups. Next, we tested for moderation of the indirect effect. Specifically, in step IV we tested whether path estimates of *T*_0_ relational victimization/conflictual teacher‐child relationship, respectively, to *T*_0_ perceived stress (paths A1 and A2) differed between the group. In step V, we tested whether the path estimate of *T*_0_ perceived stress to *T*_2_ anxiety (path B) differed between groups. Potential differences in indirect effects (step VI), were tested using the Wald test of parameter constraints (Asparouhov & Muthén, 2010). All estimates that were not part of our hypotheses (e.g., within‐time residual error correlations) were freely estimated across the two groups.

All models were fitted in Mplus version 8.0 (Muthén & Muthén, 2012), using the MLR‐estimator. Standard errors of path estimates were adjusted to account for clustering of data within schools using a sandwich estimator (Williams et al., 2000). Model fit was determined using the comparative fit index (CFI), root mean square error of approximation (RMSEA) and standardized root mean squared residual (SRMR). For the CFI, values of 0.95 and higher indicate acceptable fit and for the RMSEA and SRMR, values of 0.08 and lower indicate acceptable fit (Muthén & Muthén, 2012).

## RESULTS

### Descriptive statistics

Table 1 shows descriptive statistics. There were 207 unique air alarm soundings in the high threat region, and no occasion of air alarm in the lower threat region across the 2017–2018 period (Central Bureau of Statistics Israel, 2020). Furthermore, children in the high threat region had significant higher scores on teacher‐rated anxiety at both assessments than children in the lower threat region. No statistically significant differences between the threat regions on other study variables were found. Correlations of study variables by threat region are in Table 2.

**TABLE 1:** Descriptive statistics of sample characteristics and study variables for overall sample, and by threat region

	Overall		High threat region		Lower threat region		Test	
Rocket alarms 2017–2018 (*n*)			207		0			
Boys	%		%		%		*X* ^2^	*p*
	48		48		49		0.00	0.94

	*M*	SD	*M*	SD	*M*	SD	*F*	*p*
Age	10.61	0.78	10.70	0.93	10.53	0.62	1.39	0.16
Family size	3.02	0.93	3.02	0.92	3.03	0.94	0.10	0.86
Anxiety *T*_0_	2.78	3.38	3.15	3.56	2.19	2.85	8.71	<0.01
Anxiety *T*_1_	3.08	3.27	3.57	3.45	2.28	2.79	14.40	<0.01.
Perceived stress *T*_0_	10.68	5.64	10.56	5.83	11.06	5.55	0.69	0.41
Perceived stress *T*_1_	11.08	5.94	10.06	6.05	11.85	5.67	3.67	0.06
Relational victimization *T*_0_	1.74	1.92	1.70	2.00	1.79	1.84	0.31	0.58
Relational victimization *T*_1_	1.63	1.92	1.56	1.90	1.71	1.95	0.63	0.43
Teacher‐child conflict *T*_0_	3.73	2.69	3.51	2.65	3.92	2.73	1.68	0.09
Teacher‐child conflict *T*_1_	3.33	2.63	3.11	2.56	3.58	2.69	1.83	0.06

*Note*: Teacher‐child conflict: conflictual teacher‐child relationships. T_0_ = 2017, T_1_ = 2018.

**TABLE 2:** Correlations between study variables for high threat region (below diagonal) and lower threat region (above diagonal)

	1	2	3	4	5	6	7	8
1. Anxiety *T*_0_	‐	0.49^b^	0.16^a^	0.11	0.11	0.09	0.17^a^	0.13
2. Anxiety *T*_1_	0.59^b^	‐	0.12	0.06	0.07	0.17^a^	0.14	0.06
3. Perceived stress *T*_0_	0.18^b^	0.19^b^	‐	0.57^b^	0.34^b^	0.24^b^	0.38^b^	0.20^b^
4. Perceived stress *T*_1_	0.08	0.17^a^	0.51^b^	‐	0.26^b^	0.35^b^	0.21^b^	0.35^b^
5. Relational victimization *T*_0_	0.22^b^	0.19^b^	0.35^b^	0.16^a^	‐	0.39^b^	0.22^b^	0.08
6. Relational victimization *T*_1_	0.25^b^	0.15^b^	0.29^b^	0.29^b^	0.57^b^	‐	0.16^a^	0.19^b^
7. Teacher‐Child conflict *T*_0_	0.19^b^	0.16^a^	0.47^a^	0.21^b^	0.31^b^	0.24^b^	‐	0.52^b^
8. Teacher‐Child conflict *T*_1_	0.21^b^	0.17^a^	0.29^b^	0.38^b^	0.16^a^	0.17^b^	0.56^b^	‐

*Note*: Teacher‐child conflict: conflictual teacher‐child relationships. *T*_0_ = 2017, *T*_1_ = 2018. High threat region *n* = 220, lower threat region *n* = 188.

^a^ *p* < 0.05.

^b^ *p* < 0.01.

### Classroom psychosocial stressors, perceived stress and anxiety symptoms

The main effect model (step I) showed that *T*_0_ teacher‐child conflict was directly associated with change in anxiety symptoms from *T*_0_ to *T*_1_ (*β* = 0.06, *p* = 0.04), but relational victimization was not (*β* = 0.05, *p =* 0.38). The indirect effect model (step II), showed significant indirect effects for both psychosocial stressors (indirect effect relational victimization *β* = 0.02, *p =* 0.02; indirect effect teacher‐child conflict: *β* = 0.03, *p =* 0.03). Specifically, *T*_0_ teacher‐child conflict (*β* = 0.37, *p <* 0.001) and *T*_0_ relational victimization (*β* = 0.25, *p <* 0.001) both predicted *T*_0_ perceived stress (paths A1 and A2 of the indirect effect), which, in turn, predicted the development of anxiety from *T*_0_ to *T*_1_ (*β* = 0.09, *p =* 0.02; path B of the indirect effect). All other path estimates are in Figure 1. Model fit of the indirect effect model was adequate (CFI = 0.935; SRMR = 0.070; RMSEA = 0.092).

### Moderation by threat region

**FIGURE 2:**
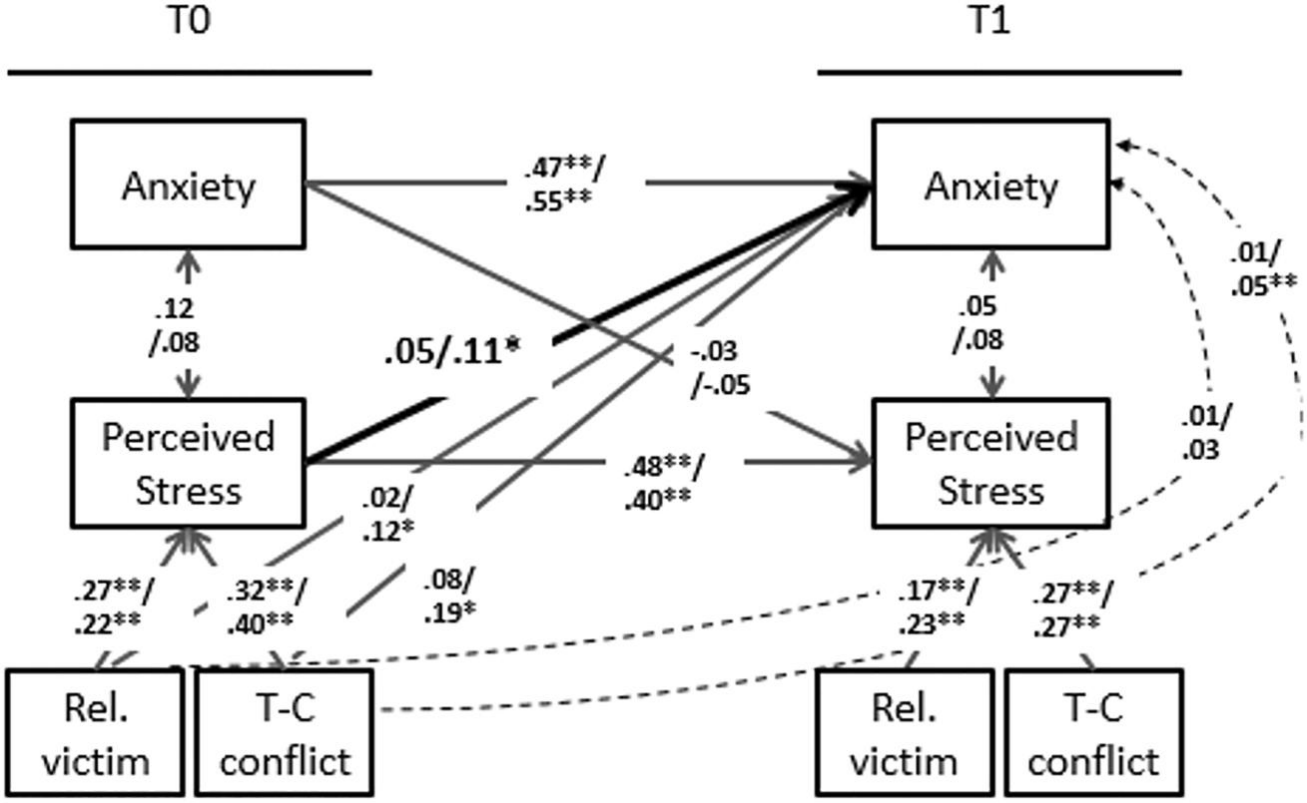
Results of path estimates in lower (upper entries) and high (lower entries) threat region. Entries in single headed arrows reflect standardized regression coefficients. Entries in double headed arrows reflect correlations coefficients. Gray entries are not significantly different between threat regions. Bold entries, of T_0_ perceived stress to T_1_ anxiety symptoms are significantly different (*p* = 0.02) between threat regions. Entries in the dotted arrow reflect the indirect effect. Rel. victim, Relational victimization; T‐C conflict, Teacher‐Child Conflict. High threat region *n* = 220, lower threat region *n* = 188. CFI = 0.99, RMSEA = 0.02 and SRMR = 0.03. *p < 0.05, **p < 0.01.

Multiple group models (lower vs. high threat region) were fitted to test for moderation by threat region. Results showed that, in the main effects model (step III), the paths of relational victimization *T*_0_ to anxiety *T*_1_ (ΔSBχ^2^(1) = 0.4, *p* = 0.15) and from teacher‐child conflict *T*_0_ to anxiety *T*_1_ (ΔSBχ^2^(1) = 1.76, *p* = .09) did not differ between the groups. Results from moderation tests of the indirect effects model showed that estimates of path A1 (relational victimization to perceived stress; ΔSBχ^2^(1) = 0.12, *p* = 0.79) and path A2 (teacher‐child conflict to perceived stress; ΔSBχ^2^(1) < 0.10, *p* = 0.90) of the indirect path (step IV) showed no differences for the two threat regions. However, estimates from perceived stress *T*_0_ to anxiety *T*_1_ (path B, step V) differed between the groups (ΔSBχ^2^(1) = 5.68, *p* = 0.01) with a significant association for children in the high threat region (*β* = 0.11, *p =* 0.02) but not for children in the lower threat region (*β* = 0.05, *p =* 0.46). Lastly (step VI), results showed that the indirect path of relational victimization/conflictual teacher‐child relationship *T*_0,_ perceived stress *T*_0_ to anxiety *T*_1_ did not differ between the groups (ΔWχ^2^(1) = 0.46, *p* = 0.74). Estimates for all other paths can be found in Figure 2.

## DISCUSSION

The aim of this study was to explore the possible exacerbating effect of living in a conflict environment on the link between the conflictual relationships with peers and teachers, perceived stress and anxiety symptoms in a sample of Israeli school children. Pertaining to our first aim, perceived stress mediated the association between classroom psychosocial stressors and anxiety development. Pertaining to our second aim, no moderation by threat‐region was found in this indirect effect. The association between perceived stress and anxiety development was only significant for children in the high threat region.

Our findings strengthened previous research on the link between perceived stress and anxiety symptoms in children and adolescents (Dimitry, 2012; Walsh et al., 2010). Previous studies on classroom stress and anxiety development did not consider war‐conflict vicinity (Dimitry, 2012; Johnsona et al., 2002; Moss, 2003; Rowe et al., 2016). We extended findings from previous studies by showing that the stress‐anxiety association depended upon chronic stress exposure associated with war‐conflict vicinity.

As predicted, the results showed an indirect path of relational victimization/conflictual teacher‐child relationship on anxiety symptoms via perceived stress. The indirect association of relational victimization/conflictual teacher‐child relationship on anxiety symptoms via perceived stress did not significantly differ between threat regions. It is possible that the differences between the threat groups in the association between perceived stress and anxiety are not strong enough to differentiate the two indirect pathways from each other. The link between perceived stress and anxiety symptoms was significant in the higher but not in the lower threat region. The moderation analysis showed a significant association for children in the high threat region but not for children in the lower threat region. In line with the cumulative risk hypothesis, perceived stress in combination with higher environmental stress constitute a risk for the development of anxiety symptoms in children in Israel.

It was also somewhat surprising to find that only mean levels of anxiety were higher in the high versus lower‐risk region, but not levels of perceived stress. It is possible that anxiety levels in the high threat region were higher due to the imminent threat experienced by children living in this region. In high threat regions, chronic exposure may lead to reduced or blunted perceived and physical stress responses in order to avoid excessive stress responses and associated detrimental health problems in a context of prolonged stress (Miller et al., 2007). However, markers of physical stress responses and information on coping abilities are needed. We would like to see our results replicated in other studies examining broader environmental sources of chronic stress, such as socioeconomic status, or stressors associated with conflict situations as in the present study.

## LIMITATIONS AND IMPLICATIONS

The results of the influence of perceived stress to anxiety should be considered with caution, as effects are small in the current study. Related to the sample studied, it needs to be tested whether our results can be generalized to other conflict areas. Furthermore, we lacked information on the experiences of family stress and socioeconomic status information of the assessed families. For example, heightened perceived and physiological stress activity has been linked to lower socioeconomic status and higher levels of maternal distress (Bates et al., 2017). Higher socioeconomic status could be expected in the lower threat region compared to the high threat region, as the average socioeconomic status in the lower threat region neighborhoods was higher (Central Bureau of Statistics Israel, 2020). The measurement of anxiety symptoms with teacher report is another limitation. By using self‐report and teacher report insights to address the stress‐anxiety link, we tested our research questions in a multiple‐informant approach design. Characteristics of teachers, such as sex and gender identity, age and teaching experiences can possibly have influenced the results. Unfortunately, we could not consider these characteristics because we did not have the according information. Conflictual relationships in the classroom and the mediator of interest, perceived stress, were both self‐report measures. Therefore, shared method variance could have influenced the observed associations.

Overall, the findings emphasize that continuous exposure to living in high threat regions makes schoolchildren increasingly vulnerable to anxiety symptoms via perceived stress in the classroom. Our results suggest that school psychologists and counselors should be particularly aware of exposure to school social stressors in children in high threat regions. Effective school‐based trauma interventions using cognitive‐behavioral techniques for children and adolescents have been designed to reduce posttraumatic stress and anxiety symptoms (Jaycox, 2004; Kataoka et al., 2003; Rolfsnes & Idsoe, 2011). Our findings suggest that related school‐based interventions should be implemented in schools in high risk regions like the Gaza vicinity. Our results also suggest exploring stressors associated with the family and the broader environment when trying to understand the association of school social stressors, perceived stress and anxiety development in elementary school aged children.

## AUTHOR CONTRIBUTIONS

All authors contributed to, read and approved the final manuscript.

## CONFLICT OF INTEREST

The authors have declared that they have no competing or potential conflicts of interest.

## ETHICS STATEMENT

Ethical approval was obtained from the Israeli Ministry of Education's ethic committee as well as from the Helsinki committee of Soroka medical center in Israel. Parents of participants provided written informed consent after procedures were fully explained.

## Data Availability

The data are not publicly available due to privacy and ethical restrictions.
